# Fevers

**Published:** 1905-05-13

**Authors:** 


### Progress in Medicine and Surgery.

### FEVERS.

(*Continued from page* 89.)

**Tick Fever.**—P. H. Ross,[32] working in conjunction with Dr. Hodges in Uganda, conceived the idea that the co-called "tick fever" might be due to a spirillum, after reading a paper by Marchaux and Salimbeni, in which they showed that a tick conveys the spirillary disease of fowls. It was found that in many cases of spirillum fever the spirilla were exceedingly rare in the blood specimen, even when taken at the height of the fever and in cases with well-marked clinical symptoms. Hence in tick fever the spirilla, if sparse, might escape notice. Subsequently spirilla were found in all of eight cases of fever in natives from whom Dr. Milne had taken blood slides. All the patients attributed their illness to the bite of ticks received in the huts of two of the native camps. The ticks, which are of a loathsome, greasy, greyish-yellow appearance, may be as large as the little finger-nail. The natives say the incubation period is from one to five days, and they avoid certain camps and localities as being hotbeds of the disease. Milne appends several charts of his patients obtained under great difficulties. The fever was brief but somewhat sharp. The symptoms of spirillum fever and tick fever are similar and consist of vomiting, diarrhœa or dysenteric stools, headache, and fever. All Milne's cases recovered with no treatment except a *placebo.*

**Cerebro-spinal Fever.**—Notes concerning an epidemic of cerebro-spinal meningitis are furnished by J. M. Ferrer.[33] The cases numbered 20, of whom 10 died. Ten cases ranged in age from three to 18 years, and 10 from 20 to 39 years. Twelve patients were males and eight females. The cases appeared after an exceptionally severe winter marked by the great prevalence and mortality of pneumonia, but it is not stated that any of the cerebro-spinal sufferers had themselves contracted pneumonia. All the patients showed in greater or less degree pain in the head, fever, rigidity of the neck, head retraction, delirium, and leucocytosis. Nearly all suffered from constipation and vomiting. Fifteen exhibited general hyperæsthesia and Kernig's sign. Stupor, coma, general rigidity and opisthotonus were frequently noted. Seven had the typical eruption, two had rose spots, and nine herpes labialis. Of complications broncho-pneumonia and "congestion of the kidneys" each occurred twice, double parotitis once. Deafness followed the attack six times. The longest case lasted 14 weeks and the shortest three days.

**Small-Pox.**—Bacteriologists have long recognised that the virulence of any particular variety of organism may be permanently or temporarily diminished by passage through a series of relatively insusceptible animals. This probably accounts for the decline in severity of a disease frequently noted towards the end of an epidemic, as has recently been demonstrated in the case of small-pox at Leicester. In this town, where infantile vaccination has been neglected recently, but where most adults over 20 have been vaccinated, a small severe epidemic occurred in 1902—18 cases with five deaths; and a much more extensive, but milder form prevailed later—700 cases after January 1903 with 25 deaths. A. Warner[34] has selected some of these mild cases for detailed description and comment. Mild cases of small-pox, varioloid, are not infrequently mistaken for chicken-pox, and they may occur not only in those persons who have been rendered partially immune by vaccination, but also in unvaccinated infants, who either enjoy a natural insusceptibility, or who have been infected with an attenuated virus. Warner calls attention to certain personal factors which may operate in the case of unvaccinated children. In Leicester he noticed that a family type of the disease prevailed, all the members of large unvaccinated families conforming to a certain type, which differed in different families even when these had all been exposed to the same original source of infection. On more than one occasion an adopted child was noted to suffer from an attack quite dissimilar from that of the rest of the family. The mother—and Ehrlich has shown that immunity is always transmitted through the maternal tissues—can confer complete immunity on her offspring, either active, which is rare, as the embryo infected with small-pox usually perishes *in utero,* or passive by transmitting the antidotal substances developed during her own illness. It seems likely, therefore, that she may also transmit a relative immunity. Cleanliness and social standing appear to give no advantage in reducing liability to infection. Stout, well-nourished people are more liable to show an abundance of typical spots. In the thin and weakly these are frequently small and abortive. The very high percentage of mild cases among those vaccinated in the incubation-period, seven or more days before the eruption appeared, shows the great importance of vaccinating contacts. Five cases of *variola sine eruptione* are reported in detail. All the patients had been vaccinated in infancy and were over 30 years of age, and the circumstances of their illness were such as to leave no reasonable doubt that they actually suffered from small-pox. Some cases without eruption have been reported also by Bancroft,[35] but these, as C. B. Ker suggests, may have been influenza. In Bancroft's report of 1,200 cases treated recently in Boston prodromal rashes were noted only 16 times. He mentions the occurrence of certain secondary rashes also, one, a mottled erythema, resembling that of scarlet fever and followed by desquamation, and the other a fine red maculo-papular eruption. The anatomy and histology of variola have been studied by Councilman, Magrath, and Brinckerhoff[36] during a recent epidemic in Boston. They describe the specific lesion of variola as a focal degeneration of stratified epithelium limited to that of the skin and of the mucous membranes of the soft palate, pharynx, and œsophagus. The lesion begins with degeneration of the deeper epidermal layers accompanied by serous and later cellular exudation. Each typical "focus" forms a fully-developed pock or pustule situated between the layers of the epidermis or between the epidermis and corium. The fluid portions are absorbed and the necrosed cellular mass enclosed between the old and the newly-formed epidermis is exfoliated by the degeneration of the former. The parasite peculiar to variola, in its cytoplasmic or its intranuclear form, is contained within these lesions, chiefly in the cells of the rete mucosum. Other changes observed in different parts of the body comprise great proliferation of cells in the hæmapoietic organs, a marked increase in the number of mononuclear basophiles in the blood and a striking decrease in the number of the polynuclear cells in the bone marrow and also in the specific lesions. The "vaccine bodies" or *cytoryctes variolæ* of Guarnieri were practically proved by Von Wasielewski in 1901 to be protozooa forming one stage in the life-cycle of a sporozoon. Another cycle takes place "within the nuclei of the generative cells of the skin, and includes all the processes of propagative reproduction." Calkins[37] has described and figured the life history of the protozoon which is analogous to that of the malarial parasite. The spores ultimately produced are exceedingly small (0·57 $\mu$). Magrath and Brinckerhoff inoculated monkeys with small-pox virus and found bodies identical with those described by Calkins in the life history of *cytoryctes variolæ* as found in men. Tyzzer[38] has done work which goes to prove that the bodies found in vaccine lymph are living organisms and the ætiological factor in the disease, and that they are identical with those found in small-pox. Recently two law suits, designed to prove the reality of the theory of aerial convection and the consequent danger of infection for the environs of small-pox hospitals, were lost. But Brinckerhoff has shown that the discs or scabs from an infected skin contain the contagium, although he has not proved that this persists on exposure to the air. Ricketts and Byles [39] have published the temperature charts and some photographs relating to the 13 cases treated by them in red light. Their results were unfavourable, although, in spite of the criticisms of the late Professor Finsen [40] on their methods, they maintain that these were calculated to invite success. The experience of J. T. C. Nash,[41] on the contrary, was favourable to the employment of red light.

[32] Brit. Med. Jour., p. 1453. [33] Med. Record, Oct. 8. [34] Practitioner, October. [35] Ibid., December. [36] Ibid. [37] Ibid. [38] Ibid. [39] Lancet, Sept. 17. [40] Ibid., Nov. 26, p. 1490. [41] Ibid.

